# Alcohol taxes’ contribution to prices in high and middle‐income countries: Data from the International Alcohol Control Study

**DOI:** 10.1111/dar.12638

**Published:** 2017-11-22

**Authors:** Martin Wall, Sally Casswell, Sarah Callinan, Surasak Chaiyasong, Pham Viet Cuong, Gaile Gray‐Phillip, Charles D. H. Parry

**Affiliations:** ^1^ SHORE & Whariki Research Centre, College of Health Massey University Auckland New Zealand; ^2^ Centre for Alcohol Policy Research, Department of Public Health, School of Psychology and Public Health La Trobe University Melbourne Australia; ^3^ Health Promotion Policy Research Centre, International Health Policy Program, Ministry of Public Health & Social Pharmacy Research Unit, Faculty of Pharmacy Mahasarakham University Mahasarakham Thailand; ^4^ Centre for Injury Policy and Prevention Research Hanoi School of Public Health Hanoi Vietnam; ^5^ National Council on Drug Abuse Prevention Basseterre Saint Kitts and Nevis; ^6^ Alcohol, Tobacco and Other Drug Research Unit South African Medical Research Council Pretoria South Africa; ^7^ South Africa and Department of Psychiatry Stellenbosch University Cape Town South Africa

**Keywords:** alcohol, excise tax, international comparison, policy, price

## Abstract

**Introduction:**

Taxation is increasingly being used as an effective means of influencing behaviour in relation to harmful products. In this paper we use data from six participating countries of the International Alcohol Control Study to examine and evaluate their comparative prices and tax regimes.

**Methods:**

We calculate taxes and prices for three high‐income and three middle‐income countries. The data are drawn from the International Alcohol Control survey and from the Alcohol Environment Protocol. Tax systems are described and then the rates of tax on key products presented. Comparisons are made using the Purchasing Power Parity rates. The price and purchase data from each country's International Alcohol Control survey is then used to calculate the mean percentage of retail price paid in tax weighted by actual consumption.

**Results:**

Both *ad valorem* and specific per unit of alcohol taxation systems are represented among the six countries. The prices differ widely between countries even though presented in terms of Purchasing Power Parity. The percentage of tax in the final price also varies widely but is much lower than the 75% set by the World Health Organization as a goal for tobacco tax.

**Conclusion:**

There is considerable variation in tax systems and prices across countries. There is scope to increase taxation and this analysis provides comparable data, including the percentage of tax in final price, from some middle and high‐income countries for consideration in policy discussion.

## Introduction

Increasing taxes on unhealthy or harmful substances is now regarded as one of the best tools to influence their use 1, 2. Taxation of alcohol has been found to be an effective and cost effective means of addressing alcohol harm through reducing consumption 3, 4. Higher excise taxes in high‐income countries have been linked to lower consumption 5, fewer alcohol‐related traffic accidents 6, fewer acute hospitalisations for intoxication 7, lower incidence of binge drinking 8 and reductions in mortality from alcohol‐related disease 9 and sudden deaths from alcohol 10. There is a much higher proportion of abstainers in middle‐income countries and men drink much more alcohol than women 11. However, a systematic review and meta‐analysis of taxation and consumption in middle‐income countries found very similar relationships to those reported in high‐income countries 12.

A tax system based solely on the absolute alcohol contained in a beverage has been discussed as the best option for public health purposes as the amount of alcohol consumed is closely linked to the extent of harm caused 13. This will work not only by reducing demand, but may also encourage producers to offer lower potency products 14. However, in all existing tax regimes the principles under which alcoholic beverages are taxed are complex and lead to differing rates of tax per unit of absolute alcohol depending on the beverage. For example, spirits are usually taxed at a much higher rate than beer or wine for a variety of reasons including the fact that spirits are cheaper to produce and so require a higher tax to keep them relatively expensive when compared to wine and beer 15.

The advantages of a specific alcohol tax linked solely to absolute alcohol content may also depend on context. Middle‐income countries have a greater proportion of abstainers than high‐income countries 16, and a tax policy which aims to protect young abstainers by ensuring higher prices for their preferred beverages may be judged to be more appropriate than a system with a sole focus on potency 17. This has been the case in Thailand 12.

Comparing countries’ excise taxation on alcohol is complicated by the fact that alcohol taxation has a long history. In many jurisdictions specific institutional or regulatory arrangements have arisen over time and remain in place. In addition to the principles outlined above, existing tax systems reflect a mixture of tradition, pragmatism, industry interests, health policy activism and protectionism.

The heterogeneity of products and contexts in which alcoholic drinks are sold and consumed makes comparisons of the impact of taxation policies on price across countries complex. This analysis is the first attempt we are aware of to calculate the strength of comparative taxation policy in this way.

This is in sharp contrast to the case of tobacco tax. The share of tax in final price has long been a policy target in tobacco control. In 1999 the World Bank set a yardstick for tobacco taxation of 67% of the final price. At the time this was the lowest level of tax as a percentage in countries where comprehensive tobacco control policies had led to reduced tobacco consumption 18. More recently the World Health Organization recommended a tax share of 75% of the final consumption price 19.

Further exploration of the existing situation with regard to alcohol taxation is urgently needed to inform discussion and policy development. This is particularly the case in middle‐income countries where the alcohol industry is aggressively expanding, which entails converting many abstainers to drinkers 20, 21.

In this paper we use data drawn from the International Alcohol Control (IAC) study, a multi‐country collaborative project to assess the impact of alcohol control policy 22. The IAC countries are largely self‐selected depending on availability of resources and this analysis included only those countries which had available data on taxation systems and prices. The study documents the nature of alcohol taxation policies and the prices paid in high and middle‐income countries. Purchasing Power Parity (PPP) rates are used to put prices and taxes into a common currency as, unlike market exchange rates, they are not affected by short term fluctuations in confidence/sentiment or monetary policy and do not only reflect goods and services traded internationally.

## Methods

### *Data sources*

The data used in this analysis drew on data from the two research tools of the IAC study. The first are survey data from drinkers, and include measures of consumption and of the prices paid for each beverage purchased. Both of these variables have been shown to be valid in analysis of New Zealand data, as indicated by the coverage achieved as compared with official statistics in the case of consumption, and with estimates of expenditure in the case of prices 23. The second tool is the Alcohol Environment Protocol (AEP) in which local researchers have provided information about their tax systems. The information in the AEP covering taxation was supplemented by searches of government websites where necessary.

### *Design and sampling*

In this article we use data collected in the first wave of the IAC survey in each country, which took place in 2011 in New Zealand, in 2012 in Thailand, in 2013 in Australia, in 2014 in Vietnam and South Africa and in 2014/16 in St Kitts and Nevis. Each country used a methodology designed to obtain random, representative samples. In Australia, a sample frame of residential landline and cell phone numbers was used. In New Zealand the sample was drawn from a frame of published and unpublished residential landline numbers. In St Kitts, Thailand, South Africa and Vietnam stratified multi‐stage methods were employed. In St Kitts and Nevis, New Zealand, Thailand and Australia respondents were drawn from the whole country, whereas the other countries concentrated on a single region or collection of regions: the Tshwane metropolitan district in South Africa and three provinces in Vietnam. Eligible participants had to be between 16 and 65 years old and have consumed alcohol in the last 6 months. Ethical approval to conduct the IAC study was obtained by each country. At the time of joining the study, none of the co‐investigators had any conflicts of interest in terms of links or funding from commercial interests involved in producing, marketing and distributing alcohol.

### *Measures*

The English IAC questionnaire was translated into and back translated from non‐English languages (Thai, Vietnamese, Afrikaans and Setswana) and piloted before use. The IAC consumption framework asks beverage specific typical quantities for each location at which participants drink. Study participants report their consumption of all beverages, including those specific to their country and informal alcohol. Respondents reported their alcohol consumption in their own terms and interviewers coded this using containers and glass sizes in which alcohol was commonly sold and served in each country.

### *Data collection*

Australia and New Zealand collected data using computer assisted telephone interviewing whereas St Kitts, Thailand, South Africa and Vietnam interviewed at the respondents’ homes. These interviews were also computer assisted using android tablets. Response rates were Australia (37%), New Zealand (60%), St Kitts and Nevis (60%), Thailand (93%), South Africa (78%) and Vietnam (99%).

### *Weighting*

As one person was selected per household, the unequal probability of respondent selection was corrected for. In South Africa, sampling weights were calculated. Where population level data relevant to sample proportions were available, post‐stratification weights were calculated (Australia and New Zealand).

### *Taxation systems and purchasing power parity*

The method of alcohol excise taxation was documented for the different countries. The taxation rates and systems were those in force during the time of each respective survey and AEP data collection. These are firstly expressed in local currency units and then inflation adjusted using the country's own Consumer Price Index to their 2011 values. They are then converted into PPP dollars. PPP rates avoid difficulties associated with the use of market exchange rates by measuring the cost of a representative basket of goods (tradable and non‐tradable) and services in different countries 24. The rates used are from the 2011 round of the World Bank's International Comparison Project 25.

### *Alcohol consumption and prices paid*

Alcohol consumption is heterogeneous. There are a number of beverages [for example, wine, beer, spirits, ready‐to‐drinks (RTD) and cider] and outlet types (off‐premise, on‐premise), each of which contains a number of sub categories. For example, within the outlet type ‘on‐premise’ there are restaurants, bars and clubs. Within the off‐premise category, supermarkets, convenience stores and liquor stores may all sell alcohol, although different jurisdictions will regulate their activities differently. There is also a wide range of packaging (bottle or can sizes and multipacks), branding and pricing options (specials, discounts and promotions) and the amount of alcohol a drink contains also can vary. Although wines and spirits tend to be of fairly uniform alcohol content, RTDs can range between 4% and 10% alcohol by volume and beer can vary in strength, although typically over a narrower range. On‐premise beer can be sold in bottles, on tap or in jugs and wine by the glass or bottle.

Each respondent in the IAC study reported alcohol consumption using a beverage and location‐specific method 26. IAC respondents who purchased alcohol were asked from which places they bought alcohol, how much they would usually buy and what would they usually pay. This was measured as the amount of local currency per ‘drink’ of alcohol (an amount of the beverage which contains 15 mL of absolute alcohol). As with taxes, prices were adjusted to 2011 values using the country's own Consumer Price Index and then converted into 2011 PPP dollars using the World Bank rates 25. We report the prices paid for each beverage‐location category, averaged across the respondents who purchased in that category and weighted by the sampling and post‐survey weights.

### *Price and tax shares*

For each individual and for each beverage the percentage of the price per unit of alcohol paid in tax was calculated using each country's schedule for excise duty. Estimates of population means and medians were then reported. We also calculated as a summary statistic the overall weighted percentage of excise tax in the final retail price across all beverages where the weights are the share of each particular beverage in each individual's reported consumption. This can be considered a comparative indicator of a country's alcohol tax stance.

## Results

### *Tax systems*

Excise taxes in this study include *ad valorem*, imposed as a percentage of the price before any sales tax is added, specific, where the tax is a predefined sum per unit of volume of the excisable good, or a mixture of the two. In some of the countries taxes are automatically indexed to maintain the influence of affordability.

### *New Zealand*

New Zealand imposes specific taxes on beer, wine and spirits with beverage and price categories set out in legislation. Specific rates are adjusted annually by the percentage increase in the Consumer Price Index over the previous 12 months. Beer and spirits are taxed per litre of absolute alcohol and wine per litre of beverage. RTDs below 6% are taxed per unit of absolute alcohol at the same rate as beer; above 6% they are taxed at a rate per litre of beverage.

### *Australia*

Australia taxes beer at a specific rate per litre of alcohol. As the first 1.15% alcohol by volume is duty free, this implies that the rate increases with the strength of the beer. Spirits are taxed at a specific rate per litre of absolute alcohol. Australia taxes wine (and some cider) using the Wine Equalisation Tax. This is imposed at a rate of 29% of the last wholesale price of the wine. The Australian Tax Office estimates this as roughly half the retail price (indicating an effective *ad valorem* rate of 14.5%) 27. However, this is only for off‐premise sales. For on‐premises, where the mark up is higher, the effective rate will be lower. Australian specific rates are inflation‐adjusted twice a year.

### *Thailand*

Thailand had (in 2013) a mixed *ad valorem* (imposed on the factory gate price) and specific tax system. When an alcoholic beverage is sold the tax is calculated using both methods and whichever is the higher is then applied. Effectively, this imposes specific taxes on high alcohol content beverages and *ad valorem* taxes on low‐alcoholic content beers and wines. This system was designed to both prevent initiation of drinking among abstainers and reduce consumption by heavier drinkers 28, 29.

### *South Africa*

South Africa had a similar excise tax regime to New Zealand with wine and cider taxed per litre of beverage (currently Rs. 3.07 per litre for table wine, Rs. 9.75 per litre of sparkling wine, Rs. 5.46 per litre of fortified wine and R2.97 per litre of cider), and beer, RTDs and spirits taxed per litre of absolute alcohol (Rs. 73.05 per litre absolute alcohol for beer, RTDs and all spirits, taxed at Rs. 149.23 per litre absolute alcohol). Traditional African (Sorghum) beer is taxed at the much lower rate of 7.82 cents per litre of beverage 30. The policy underlying the tax regime is set out in a South African Treasury document as precisely targeting the percentage of the final price that should be paid in tax (excise + VAT) at 23%, 35% and 48%, respectively of the weighted average retail price of wine, ‘clear’ (non‐traditional) beer (including ciders) and spirits. 31. The rates prevailing in 2013 are shown in Table 1 32.

**Table 1:** Specific tax rates: 2011 PPP dollars

	Australia	NZ	Thailand	SA
*Per litre absolute alcohol*				
Beer	21.48	18.76	7.85	10.49
Wine			7.85	
White spirit			11.77	
Blended spirit			27.46	
Spirits	48.87	34.16	31.39	19.73
RTDs	50.58	18.76	5.49	19.73
Cider		18.76		
*Per litre beverage*				
Beer				
Traditional beer				0.01
Wine		1.88		0.44
Sparkling wine				1.33
Fortified wine				0.81
Spirits				
RTDs				
RTDs 6%–9%		1.50		
Airag				
Cider				0.52

NZ, New Zealand; RTD, ready‐to‐drink; SA, South Africa.

### *Vietnam*

Vietnam has imposed a special consumption tax on a number of products including alcohol. This is levied at a higher *ad valorem* rate on beverages above 20% (mainly spirits) and it is also the rate charged on beer. Wine (under 20%) is taxed at a lower *ad valorem* rate. From 2012 until January 2016 the high rate was 50% and the lower rate 25%. The tax is levied on the factory gate price 33.

### *St Kitts and Nevis*

St Kitts and Nevis imposed an *ad valorem* tax on alcoholic beverages under the *Excise Act* 2010. The value of the goods on which the rate is charged is that before VAT. The rate is 15% on beer and 25% on wine and spirits. Tax becomes payable when the goods leave the manufacturer's warehouse 34.

### *Excise taxes*

Table 2 contains the rates and amounts prevailing at the time of the IAC surveys in each country. Table 1 gives these specific taxes in PPP dollars. Countries with exclusively *ad valorem* rates are not included.

**Table 2:** Tax rates/amounts in local currency units at survey date

Specific taxes	Country (local currency units)					
	AUS (AUD)	NZ (NZD)	SKN (XCD)	THAI (THB)	SA (ZAR)	VIET (VND)
*Per litre absolute alcohol*						
Beer	33.83	27.87		100	59.36	
Wine 13%				100		
RTDs	76.98	27.87		70		
Spirits > = 40%	76.98	50.76		400	111.64	
White spirits				150		
Blended spirits				350		
*Per litre beverage*						
Beer						
Traditional beer					0.08	
Wine 13%		2.787			2.5	
Sparkling wine					7.53	
Fortified wine <23%					4.59	
Cider					2.97	
RTDs		2.23^a^				
Milk alcohol (Airag)						
Spirits >40%						
*Ad valorem taxes*						
Beer			15%	55%		50%
Wine	29%		25%	60%		25%
Spirits			25%	50%		50%
Note: VAT/GST rate	10%	15%	17%	7%	14%	10%

a New Zealand taxes RTDs below 6% alcohol by volume per litre of absolute alcohol and RTDs between 6% and 9% alcohol by volume per litre of beverage.

AUS, Australia; GST, goods and services tax; NZ, New Zealand; RTD, ready‐to‐drink; SA, South Africa; SKN, St Kitts and Nevis; THAI, Thailand; VAT, value added tax; VIET, Vietnam.

Table 1 illustrates the difficulties of comparing the levels of taxation between countries, as the items on which the taxes are levied are diverse. For this reason, Table 3 shows tax amounts on internationally comparable items. The *ad valorem* taxes are based on the amount paid at the product's mean price across each country's sample. The taxes imposed on ml of absolute alcohol found in different beverages varies substantially across countries. For example, the tax on beer ranges from US$0.06 in St Kitts and Nevis to roughly five times that in New Zealand, Australia and Thailand. The highest taxes on beer are in New Zealand and on RTDs in Australia.

**Table 3:** Tax in 2011 Purchasing Power Parity US dollars on standard alcohol beverages

	Vol (mL)	Strength	Alc (mL)	Australia	NZ	SKN	Thailand	SA	Vietnam
Beer	330	5%	14.85	0.24	0.28	0.06	0.26	0.16	0.12
Wine	750	14%	101.25	1.2	1.41	1.54		0.33	
Spirits	700	40%	280	13.68	9.56	1.72	8.8	5.53	
White spirit	625	40%	250				2.9		0.31
Coloured spirit							8.2		
RTD < 6%	330	5%	14.85	0.75	0.28		0.9	0.29	
RTD > 6%	330	8%	26.4		0.50				

NZ, New Zealand; RTD, ready‐to‐drink; SA, South Africa; SKN, St Kitts and Nevis.

### *Prices*

The following Table 4 contains the price data in PPP dollars in venues where alcohol is sold for on‐ or off‐premise consumption. As with taxes, prices vary considerably across countries. On‐premise prices tend to be higher than off‐premise.

**Table 4:** US$ Purchasing Power Parity per 15 mL of alcohol 2013 (IAC Survey results)

	*N*	Mean	Median	*N*	Mean	Median
	Off‐premise			On‐premise		
*Australia*						
Beer	801	1.39	1.29	690	4.27	4.07
Wine	1033	1.29	1.19	745	4.96	4.07
Spirits	530	1.39	1.19	470	6.75	6.25
RTDs	209	1.99	1.79	143	4.07	4.37
Cider	100	1.59	1.49	73	3.77	3.67
*New Zealand*						
Beer	929	1.31	1.21	688	4.14	4.14
Wine	1419	0.91	0.71	885	4.44	4.44
Spirits	328	0.91	0.81	309	6.46	6.36
RTDs	304	1.34	1.21	148	3.84	3.94
*St Kitts & Nevis*						
Beer	**285**	1.57	1.01	711	2.75	3.00
Stout	**94**	2.05	0.91	203	2.25	2.42
Wine	130	3.34	2.13	192	6.92	6.42
Spirits	147	2.65	0.86	280	8.25	3.67
RTDs	20	3.39	2.92	37	5.50	4.83
Liqueur	64	6.13	1.55	135	7.33	6.25
Cocktails				137	14.50	11.58
*Thailand*						
Beer	812	1.65	1.76	208	3.54	2.28
White spirit	230	0.57	0.52	7	1.71	1.44
Coloured spirit	429	1.48	1.35	279	2.53	2.03
RTD/cooler				14	4.10	3.72
*South Africa*						
Beer	631	1.46	1.16	229	4.04	1.41
Wine	170	3.67	3.17	28	2.74	2.76
Spirits	174	3.30	2.67	42	7.92	4.40
RTDs	15	1.51	1.60	2		
Cider	129	2.14	1.45	89	2.84	1.92
*Vietnam*						
Beer	548	1.76	1.61	526	1.54	0.80
Light beer	85	0.99	0.75	115	1.68	1.14
Alcoholature	128	0.41	0.16	42	0.92	0.40
White spirits	579	0.42	0.25	305	0.58	0.27

IAC, International Alcohol Control; RTD, ready‐to‐drink.

### *Tax as a percentage of price*

For each IAC study country sample, the percentage of tax paid on each purchase is calculated. The consumption weighted figure in the last column (Table 5) is the percentage of the final price paid in tax averaged over all beverages, with the weights being the share of that beverage in each respondent's consumption of total absolute alcohol.

**Table 5:** Mean percentage of tax paid by beverage and outlet

	Types of Beverage					Consumption weighted
*Australia*	Beer	Wine	Spirits	RTDs	
Off	28%	14.50%	65%	51%		30%
On	9%	3.50%	17%	22%		9%
Total						28%
*New Zealand*	Beer	Wine	Spirits	RTDs		
Off	23%	43%	66%	24%		37%
On	8%	5%	10%	8%		6%
Total						27%
*St Kitts & Nevis*	Beer	Wine	Spirits	RTDs		
Off	6%	11%	11%			8%
On	4%	7%	7%			5%
Total						7%
*Thailand*	Beer	Coolers	White Spirit	Coloured Spirit		
Off	17%	18%	36%	35%		24%
On	12%	14%	29%	24%		19%
Total						21%
*South Africa*	Beer	Wine	Spirits	Cider		
Off	16%	7%	43%	12%		20%
On	13%	4%	19%	8%		11%
Total						19%
*Vietnam*	Beer	Light beer	Spirits	Alcoholature	White spirit	
Off	15%	15%		0%	0%	7%
On	10%	10%	10%	0%	0%	5%
Total						7%

RTD, ready‐to‐drink.

## Discussion

The descriptions of the tax systems in this paper illustrate the diversity in place in different jurisdictions. The countries considered here mainly tax alcohol using purely *ad valorem* or purely specific excise taxes. The exception is Thailand where the tax system at the time of the study was designed to address the policy aim of maintaining the high rate of 60% abstention. Australia also has an *ad valorem* wine and cider tax whereas all its other rates are specific.

The approach taken here allows comparisons to be made in a consistent fashion about the strength of a country's tax policy with regard to alcohol. We have shown that consumers in New Zealand pay about 37%, Australia 30%, Thailand 24% and South Africa 20% of the final price in excise tax on alcohol purchased from off‐premise, whereas in Vietnam and St Kitts the rate is 7%–8%. The lower rate for Australia compared with New Zealand is due to the low taxes imposed on wine (and cider) through the Wine Equalisation Tax, described by the Henry Review as ‘currently designed as a value‐based revenue‐raising tax, is not well suited to reducing social harm’ 35.

The percentage of final price paid in tax in some of countries is much lower for on‐premise than for off‐premise locations. For example, 11% on versus 20% off for South Africa, 9% versus 30% for Australia and 6% versus 37% for New Zealand. This is because of the higher prices charged in on‐premise as opposed to off‐premise locations. Off‐premise and on‐premise prices differ more in high‐income countries where there is often a strong legal distinction between selling alcohol on‐ or off‐premise compared to some middle‐income countries where the demarcation is not as important. For example, in Thailand the average consumption weighted tax percentage is 19% for on‐premise alcohol as compared with 24% for off‐premise.

South Africa has a particular tax pattern with on‐premise tax percentages similar to Australia and New Zealand, but lower off‐premise tax percentages at 20% when compared to the other middle and high‐income countries. South African policy makers have a stated aim of setting alcohol taxation to reflect international norms, whilst at the same time limiting the incentives to consume illicitly produced or traded alcohol with its associated health risks 31.

Much Vietnamese consumption is in the form of untaxed informal beverages and so the overall low weighted tax percentage is a result of consumers paying zero tax on much of what they drink.

These results indicate one aspect of the complexity of assessing the percentage of retail price of alcohol compared with tobacco given the differences in on‐premise and off‐premise prices. The taxes also vary by beverage adding a further level of complexity. However, these results provide some initial information to compare taxation goals between alcohol and tobacco. The World Health Organization has recommended that for effective tobacco control, the share of tax in the final retail price of tobacco should be at least 75%. In 2015, the year that the recommendation was made, taxation was highest in the high‐income countries with 35% of these countries meeting the 75% target, whereas only 10% of middle‐income and about 3% of low‐income countries achieving tax at or above 75% of retail price 19. However, these levels are well in excess of the percentages found in this study for alcohol.

This tobacco tax target or yardstick provides a simple measure of the extent to which tobacco taxation is at levels which are sufficient to reduce consumption. This approach has created an easy to understand method by which countries can ‘benchmark’ their tax incidence against global best practice. However, it has limitations in the context of low‐ or middle‐income countries with rapidly expanding economies in which case affordability needs to be taken into account 36. However in the context of a far less advanced research and policy literature on alcohol taxation, the measures provided in this paper are useful illustrations of the target based on tax as a percentage of retail price.

## Conclusion

Alcohol taxes are an effective tool for policymakers to reduce alcohol consumption and harm. In practice they are highly heterogeneous as country taxation systems have developed to meet a range of objectives over a long period of time. Calculations of the proportion of retail price contributed by taxation provide a benchmark for countries and can inform policy debate.
